# The Role of Cumulative LDL Cholesterol in Cardiovascular Disease Development in Patients with Familial Hypercholesterolemia

**DOI:** 10.3390/jpm12010071

**Published:** 2022-01-07

**Authors:** Victoria Korneva, Tatyana Kuznetsova, Ulrich Julius

**Affiliations:** 1 Faculty Therapy Department, Petrozavodsk State University, Lenin Avenue, 33, 185000 Petrozavodsk, Russia; eme@karelia.ru; 2 Lipidology and Lipoprotein Apheresis Center, Department of Internal Medicine III, University Hospital Carl Gustav Carus, Technische Universität Dresden, Fetscherstr. 74, 01307 Dresden, Germany; ulrich.julius@ukdd.de

**Keywords:** familial hypercholesterolemia, cumulative LDL-C level, prognosis

## Abstract

In patients with familial hypercholesterolemia (FH) the exposure of very high LDL-C concentration and cumulative LDL-C level (cum LDL-C) can play a significant role in the prognosis. Objective: to analyze the contribution of “cum LDL-C for all life” and the index “cum LDL-C/age” to the development of coronary heart disease (CHD), myocardial infarction (MI), and a combined end point: MI, stroke, unstable angina in FH patients. Methods: 188 patients (mean age 49.2 years, males 45.7%) with FH were examined (Dutch Lipid Clinic Criteria). We had evaluated cumulative LDL-C and index “cum DL-C/age” along with other classical risk factors. Cum LDL-C was calculated as LDL-Cmax × (age at initiating of hypolipidemic therapy) + LDL-C at inclusion age at initiation/correction therapy). Cumulative LDL-C and “cum LDL-C/age” were calculated as the ratio cum LDL-C to age. The follow-up period was 5.4 (from 3 to 10) years. Results: The index “cum LDL-C/age” was higher in patients with CHD 58.7 ± 10.4 mmol/L/years vs. 40.1 ± 11.7 mmol/L/years in patients without CHD (*p* < 0.001). According to our data based on the results of the logistic regression analysis in patients with FH, cumulative LDL-C and the cumulative index “cum LDL–C/age” played a strong predictive role in the development of CHD in FH patients; it was greater than the role of TC and LDL-C concentrations. We present ROC curves for CHD, MI and combined end point in FH patients, and a prognostic scale for CHD development, which is based on classical cardiovascular risk factors. Conclusion: cumulative LDL-C level plays an important role in the development of CHD in FH patients.

## 1. Introduction

Familial hypercholesterolemia (FH) is an autosomal codominant genetic disease, which is characterized by an elevation of low-density lipoprotein cholesterol (LDL-C) and a deposition of LDL-derived cholesterol in tendons (xanthomatosis) and arteries, and which leads to premature cardiovascular disease [1,2,3,4]. However, cardiovascular risk among these patients can vary widely, even in those who belong to the same family or share the same mutation [5,6,7]. FH patients refer to the high and very high cardiovascular risk categories, but scales, such as SCORE and the Framingham Risk Score, cannot be applied in these subjects [8,9]. Classic cardiovascular risk factors can contribute with high variability to the presence of cardiovascular disease. Although the use of statins reduces the cardiovascular risk, FH patients have still a high residual risk [5]. In patients with FH the exposure of very high LDL-C concentration since birth [10] and cumulative LDL-C level (cum LDL-C) can play a significant role in the prognosis of the disease. 

## 2. Aim

To analyze the contribution of “cum LDL-C for all life” and the index “cum LDL-C/age” to the development of coronary heart disease (CHD), myocardial infarction (MI), and a combined end point (MI, stroke, unstable angina) in addition to classic cardiovascular risk factors in FH patients.

## 3. Methods

One hundred and eighty-eight patients (mean age 49.2 (range 18–64) years, males 86 (45.7%) with FH were examined (Dutch Lipid Clinic Criteria). In 118 patients, a definite FH was diagnosed (62.7%), a genetic test was performed in 102 patients (54.2%). We had evaluated cumulative LDL-C and index “cum LDL-C/age” along with classical risk factors (total cholesterol (TC), LDL-C, HDL-C, triglycerides (TG), body mass index (BMI), burdened heredity for cardiovascular diseases, age, gender, smoking). For calculation of cumulative LDL-C we used formula: Cum LDL-C = LDL − Cmax × age at initiation of hypolipidemic therapy + LDL-C at inclusion-age at initiation/correction therapy) [11]. Index cumulative LDL-C and index “cum LDL-C/age” were calculated as ratio cum LDL-C to age of patients. 

Additional laboratory criteria were lipoprotein(a) (Lp(a)), creatinine and glomerular filtration rate calculated according to CKD-EPI formula. In the follow-up period of 5.4 (from 3 to 10) years, we analyzed the development of CHD, MI and calculated the combined end point (CEP). CHD was diagnosed according to typical clinical symptoms (angina pectoris), ischemic criteria on ECG monitoring and stress test. If there were indications for a coronary angiography this was performed. MI was diagnosed in the presence of medical history data, ECG chance and positive troponin test. 

Patients who stopped smoking were considered non-smokers.

Positive family history was considered if acute coronary syndromes were also observed in relatives of men under the age of 55 and woman under the age of 65.

Arterial hypertension was diagnosed when blood pressure was detected above 140 and 90 mmHg before the start of therapy. Further, the patient was considered to have arterial hypertension, despite the blood pressure figures on the background of treatment. 

The study was conducted according to the guidelines of the Declaration of Helsinki, and approved by Ethics Committee of the Ministry of Health and Petrozavodsk State University (date of approval 14 November 2013), protocol code No. 29. 

All patients included in the study signed informed consent. The principles of the study comply with the Helsinki Declaration.

For 15 years we have maintained a register of FH patients in Karelia Republic. (It now includes 380 patients). All patients have been observed by us on the basis of the lipid center created at the medical institute of Petrozavodsk state university. We had two sources for our register: the first one was patients who were treated in emergency care hospital in Petrozavodsk (28,225 patients) and the second was patients with severe dyslipidemia, who were forwarded to our clinic by outpatient cardiologists for special consultation.

## 4. Results

Non-lipid risk factors in FH patients with CHD are presented in Table 1. Lipid risk factors in FH patients with CHD are given in Table 2. FH patients with CHD were older than patients without CHD: mean age was 58.7(range 34–84) years and 40.1 (range 18–64) years, respectively (*p* < 0.0001).

TC concentrations in FH patients with CHD were 9.9 ± 1.8 mmol/L, in patients without CHD 9.3 ± 1.8 mmol/L; *p* = 0.003. LDL-C in CHD patients was 7.2 ± 1.6 mmol/L, without CHD 6.6 ± 1.2 mmol/L; *p* = 0.004. TG in CHD patients was 1.79 ± 0.6 mmol/L vs. 1.48 ± 0.6 mmol/L; *p* = 0.004. HDL-C in CHD was 1.4 ± 0.4 mmol/L vs. 1.7 ± 0.5 mmol/L, *p* = 0.0007. The mean age at the start of a hypolipidemic therapy in FH patients with CHD was 54.2 ± 10.0 years vs. 42.2 ± 10.3 years without CHD, *p* < 0.001. Cum LDL-C in FH patients with CHD was 409.4 ± 126.3 mmol/L × age vs. 261.1 ± 85.8 mmol/L × age, *p* < 0.001. The index “cum LDL-C/age” was higher in patients with CHD 58.7 ± 10.4 mmol/L/years vs. 40.1 ± 11.7 mmol/L/years in patients without CHD (*p* < 0.001).

Glucose concentrations in patients with CHD were higher: 5.4 ± 1.9 mmol/L vs. 4.9 ± 0.6 mmol/L, *p* = 0.03. BMI in CHD patients was higher: 28.2 ± 3.9 kg/m^2^ vs. 26.5 ± 3.7, *p* = 0.005; the waist size (WS) in CHD was 87.7 ± 12 cm vs. 75.2 ± 12 cm (*p* < 0.001). Additionally, 85.2% of CHD patients had a hypertension vs. 41.9% of patients without CHD, *p* < 0.0001. A positive family history of cardiovascular diseases was found in 80 (78.4%) of patients with CHD compared to 50 (58.1%) in patients without CHD, *p* = 0.005.

The creatinine concentration in patients with CHD was 92.1 ± 20 umol/L, in patients without CHD it was 76.3 ± 14.2 (*p* < 0.001) and the glomerular filtration rate (GFR) according to the CKD-EPI formula was lower in FH patients with CHD: 70.2 ± 17.1 mL/min/1.73 m^2^ vs. 100.1 ± 17.4 mL/min/1.73 m^2^, *p* < 0.001).

It was found by logistic regression that the cum LDL-C level and the index “cum LDL-C/age” had a greater prognostic value for the development of CHD in FH patients compared to TC and LDL–C concentrations. Other significant prognostic factors included gender, TG initial, hypertension and positive family history of CHD (Table 3).

**Figure 1:**
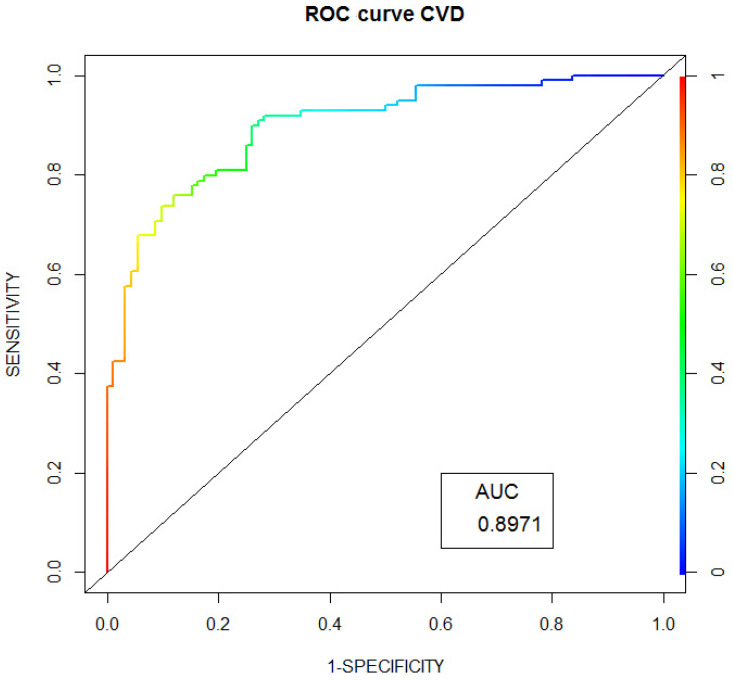
ROC curve in the prognostic model of the coronary heart disease (CHD) outcome when taking into account the index “cum LDL-C” in FH patients.

Thus, the cumulative level of LDL-C plays an important prognostic role in the CHD development along with classic risk factors. Figure 1 shows the ROC curve in the prognostic model of the CHD outcome in patients with complex risk factors, including cum LDL-C.

The characteristics of non-lipid risk factors in FH patients with MI are presented in Table 4, and lipid factors are presented in Table 5.

In patients with MI the Lp(a) level was 0.67 ± 0.7 g/L, compared with 0.3 ± 0.42 mmol/L in patients without MI, *p* = 0.008. The TC and LDL-C concentrations were not significantly different in patients with and without MI. It should be noted that although the age of lipid-lowering therapy initiation in both groups did not differ, in patients with MI the lipid-lowering therapy was less intense. The percentage of TC and LDL-C reduction was 17.0 ± 22% and 21.0 ± 27.0% in patients with MI after 1 year, and 25.3 ± 23.6% (*p* = 0.047) and 31.5 ± 29.9% in patients without MI (*p* = 0.048). Gender differences were found between these subgroups: the number of men was more 31 (59.6%) in patients with MI than 47 (37.3%) in patients without MI, *p* = 0.007. 

A positive family history of CHD was found in 52 (100%) patients with MI and 71 (59.1%) in patients without MI, *p* = 0.001.

Creatinine concentrations in patients with MI were significantly higher: 96.2 ± 22.3 umol/L vs. 79 ± 15 (*p* = 0.0002). The GFR according to the CKD-EPI formula was lower 70.4 ± 17.7 mL/min/1.73 m^2^ in patients with MI compared with 90 ± 23 mL/min/1.73 m^2^ in FH patients without MI, *p* = 0.0003. Additionally, among patients with MI the number of smokers was significantly higher: 22 (43.1%) compared with 22 (18.2%) in patients without MI, *p*= 0.001.

The logistic regression shows the most significant factors which determine the probability of MI in patients with FH: positive family history of CHD, gender, initial TC level, cum LDL-C/age, the presence of hypertension and smoking (Table 6).

**Figure 2:**
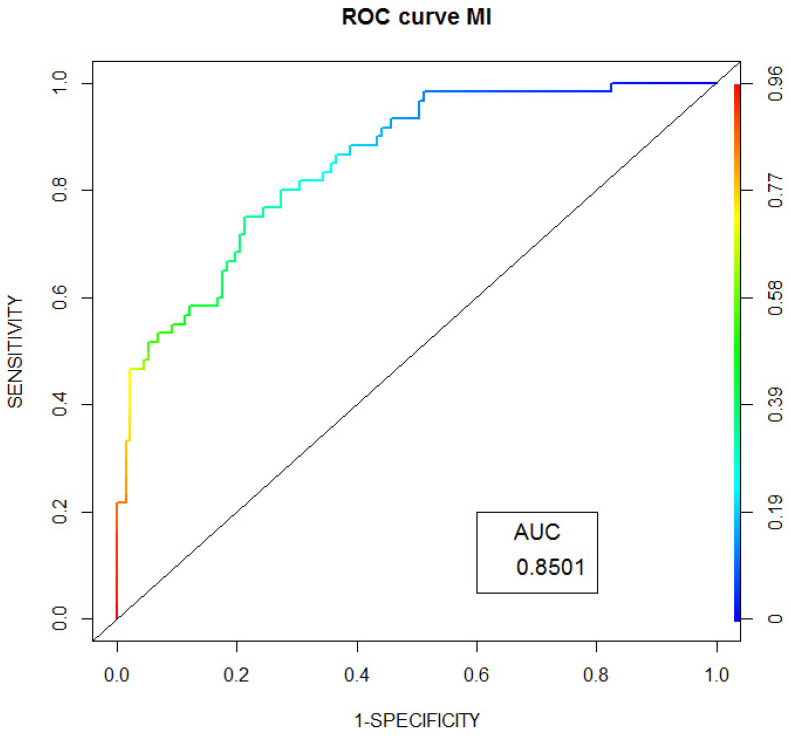
ROC curve of prognostic model of MI outcome in FH patients.

Figure 2 shows the ROC curve in the prognostic model of MI outcome in FH patients. 

The combined end point (CEP) included MI, unstable angina, and ischemic stroke.

Non-lipid risk factors in patients with CP and FH are presented in Table 7; the lipid factors are presented in Table 8.

In patients with CEP, the Lp(a) level was higher: 0.63 ± 0.59 g/L compared with 0.29 ± 0.42 g/L without CP, *p* = 0.001. A similar trend was noted for the TC and LDL-C concentrations. HDL-C concentrations were higher in patients without CEP: 1.6 ± 0.5 mmol/L compared with 1.5 ± 0.5 mmol/L in patients with CEP (*p* = 0.040). The cum LDL-C was significantly higher in FH patients with CEP: 397.8 ± 125.4 mmol/L × years compared to 309.2 ± 124.8 without CEP (*p* < 0.001). The ratio “cum LDL-C/age” was also higher in patients with CP: 57.4 ± 11.0 vs. 46.1 ± 14.5 (*p* < 0.001).

It should be noted that the age of lipid-lowering therapy initiation in both groups did not differ; however, the lipid-lowering therapy in patients with CEP was started later, and it was more intensive (the percentage of TC reduction was 28.3 ± 23.9% compared to 14.7 ± 20.6%, *p* = 0.00006). A similar trend was observed for the LDL-C level: 34.7 ± 30.0% in patients with CEP and 18.3 ± 25.6% in patients without CEP (*p* = 0.0001).

The age of CHD onset in patients without CEP was higher, at 58.1 ± 7.5 years compared to 51.1 ± 10.3 in patients with CEP (*p* < 0.001). There were gender differences between the subgroups with and without CEP; the number of men was equal to 55.9% in patients with CEP and to 39.2% in patients without CEP, *p* = 0.028.

Positive family history of CHD was found in more patients with CEP: 60 (88.2%) vs. 69 (57.5%), *p* < 0.0001. The BMI did not differ between the groups; the waist size was significantly higher in patients with CEP: 87.5 ± 11.9 cm vs. 79.9 ± 13.6 (*p* = 0.007).

The creatinine concentration in patients with CEP was significantly higher: 93.4 ± 20.6 umol/L vs. 78.9 ± 15.6 umol/L (*p* < 0.001), and GFR (CKD-EPI formula) was 71.6 ± 17.0 mL/min/1.73 m^2^ in patients with CEP, and 91.6 ± 22.6 mL/min/1.73 m^2^ in patients without CEP, *p* < 0.001. There was a significantly higher number of smokers among patients with CEP: 30 (44.1%) vs. (18.3%), *p* = 0.0003. The number of patients with hypertension was higher in patients with CEP: 58 (85.2%) vs. 64 (53.3%) in patients without CEP, *p* < 0.0001.

The logistic regression shows the most significant factors which determine the probability of CEP in patients with FH: positive family history of IHD, gender (male), age, hypertension and smoking (Table 9).

**Figure 3:**
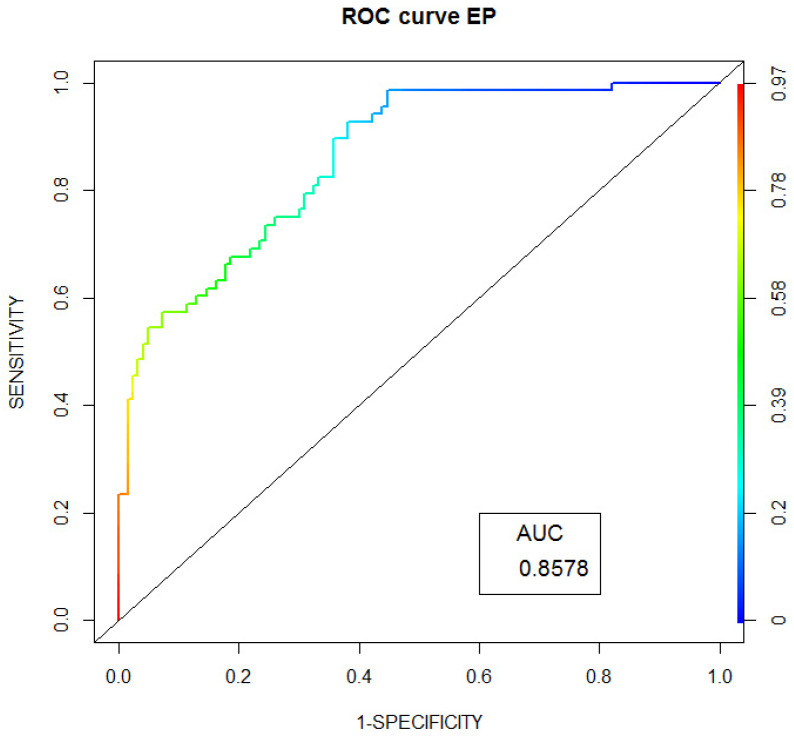
ROC curve in the prognostic model of CEP outcome in FH patients.

Figure 3 shows ROC curve in the prognostic model of CEP outcome in FH patients.

The scale for assessing the risk of a combined end point developing in patients with certain and probable FH was developed using a prognostic formula, obtained by step-by-step logistic regression. All factors are available for evaluation in general clinical practice: age, gender, smoking, burdened heredity for cardiovascular diseases and the presence of arterial hypertension. The scale is presented separately for men and women. Of course, genetic analysis is an important prognostic factor in FH patients, but in real clinical practice a doctor usually only has information about the phenotypic manifestation of the disease.

Table 10 and Table 11 are showing the scale for assessing the risk of CEP in patients with defined and probable FH for men and women.

Based on gender and age, one must look at the appropriate verse in Table 10 or Table 11 and ask whether the patient had the following three risk factors: positive family history, hypertension and smoking. Then evaluate the risk of combined end point developing in FH patients.

### Limitations

The number of patients including in this study was not too big. The analysis performed validates a CV risk equation in a population of FH patients with either genetic or clinical diagnosis of FH. The study included only individuals living in northwestern part of Russia. The lack of a control group of patients with CV events and not affected by familial hypercholesterolemia can limit the strength of the findings.

## 5. Discussion

Classical cardiovascular risk factors and the Lp(a) concentration in FH patients play an important role in prognosis, but their contribution in FH may differ from the general population. Besides the genetic nature of the disease, other risk factors contribute, such as cumulative LDL levels. It was shown that LDL-C levels are more important than the type of mutation, reinforcing the concept that the phenotype is more important than the genotype in the risk of patients with familial hypercholesterolemia [12].

According to our data based on the results of the logistic regression analysis in patients with FH, cumulative LDL-C and the cumulative index “cum LDL-C/age” played a strong predictive role in the development of CHD in FH patients; it was greater than the role of TC and LDL-C concentrations.

Several predictive scales for FH patients were suggested, such as the Montreal—FH-Score and SAFEHEART registry; however, they were based only on classical risk factors (age, HDL-C, gender, hypertension and smoking). It is also important to take into account the indicator “cumulative level of LDL-C” for patients with FH [13,14].

The role of cum LDL-C was discussed in the literature [11,15]. The cholesterol-year-score is a simple tool which evaluates the duration and intensity of vascular exposure to elevated cholesterol levels [16], and it is a similar to that in which pack-year is used to measure lifelong exposure to tobacco [11]. An increased prevalence of early atherosclerotic burden has been observed in young FH subjects who present a high vascular cholesterol burden [17]. The cholesterol-year-score was first used in homozygous FH subjects [16] but its validation in FH patients has not been reported to date [11].

Early initiating of lipid-lowering therapy is very important. The cumulative burden of 6250 mg-years of LDL by age 50 years means that person has very likely already developed a large atherosclerotic plaque burden. As a result, lowering LDL-C after this cumulative exposure can reduce the risk of cardiovascular events, but this person will remain at relatively high “residual” risk of experiencing an acute cardiovascular event because one of the existing plaques can still disrupt and cause an acute coronary syndrome [15]. After the cumLDL-C exposure threshold has been exceeded, the incidence of MI appears to double with each increasing decade of exposure to the same plasma level of LDL-C. For example, the risk of MI increases from 1% after 5000 mg-years (129.2 mmol-years) of cumulative exposure to LDL-C by age 40 years, to 2% after 6250 mg-years (156.3 mmol-years) of exposure by the age 50 years, to 4% after 7500 mg-years (187.5 mmol-years) of exposure by the age 60 years [14].

In the study of Gallo A. the LDL-year score was a strong predictor of CV events in FH patients, especially in primary prevention. The CV risk is multiplied by 5.7-fold for patients with a cholesterol-year-score between 6000 and 16,000 mg/dL and by 17.4 for those with a cholesterol-year-score above 16,000 mg/dL. More interestingly, we observed that a LDL-C-year-score above 6000 mg/dL provided a similar CV risk to that in patients who had already suffered from a CV event, suggesting that earlier initiation of lipid lowering therapy might contribute to reduction in the incidence of CV events [16].

## 6. Conclusions

The “cum LDL-C” level plays a more important role in the development of CHD in FH patients than LDL-C. The index “cum LDL-C/age” has a still higher significance in the development of MI in FH patients, than LDL-C.

We present ROC-curves for CHD, MI and CEP in FH patients. We also present a prognostic scale for CHD development, which is based on classical cardiovascular risk factors.

## Figures and Tables

**Table 1:** Non-lipid risk factors and CHD development in FH patients.

	With CHD (*n* = 102)	Without CHD (*n* = 86)	*p*
Age, years	58.7 ± 10.4	40.1 ± 11.7	<0.0001
Males, *n* (%)	44 (43.1%)	42 (48.8%)	0.436
Creatinine, umol/L	92.1 ± 20	76.3 ± 14.2	<0.001
Glomerular Filtration Rate (GFR, CKD-EPI), ml/min/1.73 m^2^	70.2 ± 17.1	100.1 ± 17.4	<0.001
Glucose level, mmol/L	5.4 ± 1.9	4.9 ± 0.6	0.03
BMI, kg/m^2^	28.2 ± 3.9	26.5 ± 3.7	0.005
Waist size, cm	87.7 ± 12	75.2 ± 12	<0.001
Hypertension, *n* (%)	87 (85.2%)	36 (41.9%)	<0.0001
Smoking, *n* (%)	33 (32.4%)	19 (22%)	0.153
Positive family history, *n* (%)	80 (78.4%)	50 (58.1%)	0.005

The data are presented in the form of mean and standard deviation.

**Table 2:** Lipid risk factors and CHD development in FH patients.

	With CHD (*n* = 102)	Without CHD (*n* = 86)	*p*
Lp(a), g/l	0.55 ± 0.5	0.27 ± 0.4	0.005
Total Cholesterol (TC) initial, mmol/L	9.9 ± 1.8	9.3 ± 1.2	0.003
Low density cholesterol (LDL-C) initial, mmol/L	7.2 ± 1.6	6.6 ± 1.2	0.004
High density cholesterol (HDL-C) initial, mmol/L	1.4 ± 0.4	1.7 ± 0.5	0.0007
Triglycerides (TG) initial, mmol/L	1.8 ± 0.7	1.5 ± 0.7	0.004
TC on treatment, mmol/L	7.2 ± 2.3	8.1 ± 1.9	0.004
LDL-C on treatment, mmol/L	4.8 ± 2.1	5.6 ± 1.7	0.008
HDL-C on treatment, mmol/L/	1.4 ± 0.4	1.6 ± 0.5	<0.001
TG on treatment, mmol/L	2.1 ± 1.3	1.5 ± 0.6	<0.001
Percentage of TC reduction, %	26.6 ± 22.9	11.9 ± 20.1	<0.001
Percentage of LDL-C reduction, %	32.9 ± 28.9	14.7 ± 24.3	<0.001
Cum LDL-C, mmol/L × years	409.4 ± 126.3	261.1 ± 85.8	<0.001
Index “cum LDL-C/age”, mmol/L × years	58.7 ± 10.4	40.1 ± 11.7	<0.001
Age at hypolipidemic therapy start, years	54.2 ± 10.0	42.2 ± 10.3	<0.001

The data are presented in the form of means and standard deviation.

**Table 3:** Statistical significance of coefficients in calculating the odds ratio of IHD in FH patients (logistic regression analysis).

Predictors	Coefficient	Statistical Error	OR	*p*
Deviation	−4.66	1.47	0.009	0.0015
Positive family history of CHD	1.46	0.45	4.35	0.001
Male gender	0.97	0.44	2.64	0.027
Cum LDL-C	0.018	0.003	1.02	<0.0001
Cum LDL/age	−0.71	0.24	0.49	0.003
TG initial	0.75	0.30	2.12	0.011
Hypertension	0.77	0.44	2.16	0.084

Formula for calculating OR and probability of CHD: OR(CHD) = exp(−4.66 + 1.46 × Family history + 0.97 × male sex + 0.018 × cum LDL-C − 0.71 × cum LDL-C/age + 0.75 × TG initial + 0.77 × Hypertension. *p* (CHD) = 1/(1 + (1/OR)). The threshold classification = 0.45. If *p* (CHD) < 0.45, the absence of CHD is predicted. If *p* (CHD) ≥ 0.45, CHD is predicted. At the accepted threshold, the sensitivity is 84.8%; specificity is 75.0%.

**Table 4:** Non-lipid risk factors and MI in FH patients.

Predictors	With MI (*n* = 52)	Without MI (*n* = 126)	*p*
Age, years	56.9 ± 11.9	47 ± 15	0.987
Males, *n* (%)	31 (59.6%)	47 (37.3%)	0.007
Creatinine, umol/L	96.2 ± 22.3	79 ± 15	0.0002
GFR, ml/min/1.73 m^2^	70.4 ± 17.7	90 ± 23	0.0003
Glucose, mmol/L	5.0 ± 2.0	5.5 ± 1.2	0.172
BMI, kg/m^2^	28.2 ± 37	27.0 ± 4.0	0.225
Waist size, cm	88.0 ± 11.9	80.0 ± 14.0	0.038
Age of onset of CHD, years	51.7 ± 0.18	58.0 ± 8.0	0.013
Hypertension, *n* (%)	41 (78.8%)	89 (70.6%)	0.063
Smoking, *n* (%)	22 (43.1%)	22 (18.2%)	0.001
Positive family history of CHD, *n* (%)	52 (100%)	71 (59.1%)	0.001

The data are presented in the form of average and standard deviation.

**Table 5:** Lipid risk factors and MI in FH patients.

Predictors	With MI (*n* = 52)	Without MI (*n* = 126)	*p*
Lp(a), g/L	0.67 ± 0.7	0.3 ± 0.42	0.008
TC initial, mmol/L	9.6 ± 1.7	10.0 ± 1.0	0.987
LDL-C initial, mmol/L	6.9 ± 1.4	7.0 ± 1.0	0.151
HDL-C initial, mmol/L	1.5 ± 0.5	2.0 ± 0.1	0.318
TG initial, mmol/L	1.8 ± 0.6	2.0 ± 1.0	0.574
TC on treatment, mmol/L	7 ± 2.2	8.0 ± 2.0	0.055
LDL-C on treatment, mmol/L	4.7 ± 2.0	5.0 ± 2.0	0.146
HDL-C on treatment, mmol/L/	17.0 ± 22	25.3 ± 23.6	0.047
TG on treatment, mmol/L	1.9 ± 0.7	2.0 ± 1.0	0.464
Percentage of TC reduction, %	17 ± 22	25.5 ± 14.6	0.048
Percentage of LDL-C reduction, %	46.8 ± 14.6	56.9 ± 11.9	<0.001
Cum LDL-C, mmol/L × years	50.0 ± 12.0	52.6 ± 10.3	0.500

The data are presented in the form of means and standard deviation.

**Table 6:** Statistical significance of coefficients in calculating the odds ratio of MI in FH patients (logistic regression).

Predictor	Coefficient	Statistical Error	OR	*p*
Deviation	−4.59	1.34	0.01	0.0006
Positive family history of CHD	1.69	0.46	5.42	0.0002
Male gender	1.11	0.43	3.03	0.010
TC initial	0.35	0.19	1.41	0.061
Cum LDL-C/age	−0.43	0.22	0.65	0.051
Hypertension	1.75	0.44	5.75	<0.0001
Smoking	1.07	0.44	2.92	0.015

Formula for calculating OR and MI probability: OR(MI) = exp − 4.59 + 1.69 × Family history + 0.35 × TC − 0.43 × cum LDL-C/age + 1.75 × Hypertension + 1.07 × smoking. P(MI) = 1/(1 + (1/OR)). The threshold classification = 0.19. If *p* (MI) < 0.19, MI is not predicted. If *p* (MI) ≥ 0.19, the MI is predicted. At the accepted threshold, the sensitivity is 81.8%; the specificity is 70.7%.

**Table 7:** Assessment of the global non-lipid risk factors impact for the development of the combined end point (CEP).

	With CEP (*n* = 68)	Without CEP (*n* = 120)	*p*
Age, years	57.4 ± 11.0	46.1 ± 14.5	<0.001
Males, *n* (%)	38 (55.9%)	47 (39.2%)	0.028
Creatinine, umol/L	93.4 ± 20.6	78.9 ± 15.6	<0.001
GFR, mL/min/1.73 m^2^	71.6 ± 17.0	91.6 ± 22.6	<0.001
Glucose, mmol/L	5.4 ± 1.1	5.0 ± 1.7	0.128
BMI, kg/m^2^	28.1 ± 4.1	27.0 ± 3.7	0.084
Waist size, cm	87.5 ± 11.9	79.9 ± 13.6	0.007
Age of CHD onset, years	51.1 ± 10.3	58.1 ± 7.5	<0.001
Hypertension, *n* (%)	58 (85.2%)	64 (53.3%)	<0.0001
Smoking, *n* (%)	30 (44.1%)	22 (18.3%)	0.0003
Positive family history of CHD, *n* (%)	60 (88.2%)	69 (57.5%)	<0.0001
CHD, *n* (%)	67 (98.5%)	34 (28.3%)	<0.0001

The data are presented in the form of means and standard deviation.

**Table 8:** Lipid risk factors in FH patients with combined end point (CEP).

	With CEP (*n* = 68)	Without CEP (*n* = 120)	*p*
Lp(a), g/L	0.63 ± 0.59	0.29 ± 0.42	0.001
TC initial, mmol/L	10.0 ± 1.9	9.4 ±1.3	0.022
LDL initial, mmol/L	7.2 ± 1.6	6.8 ± 1.3	0.030
HDL initial, mmol/L	1.5 ± 0.5	1.6 ± 0.5	0.040
TG initial, mmol/L	1.8 ± 0.6	1.6 ± 0.7	0.293
TC on treatment, mmol/L	7.0 ± 2.2	8.0 ± 2.0	0.002
LDL-C on treatment, mmol/L	4.7 ± 2.1	5.4 ± 1.8	0.012
Percentage of TC reduction, %	28.3 ± 23.9	14.7 ± 20.6	0.00006
Percentage of LDL-C reduction, %	34.7 ± 30.0	18.3 ± 25.6	0.0001
Cum LDL-C, mmol/L × years	397.8 ± 125.4	309.2 ± 124.8	<0.001
Index “Cum LDL-C/age”, mmol/L × years	57.4 ± 11.0	46.1 ± 14.5	<0.001
Age of hypolipidemic therapy start, years	52.5 ± 10.5	49.8 ± 12.0	0.173

The data are presented in the form of average and standard deviation.

**Table 9:** Statistical significance of coefficients in calculating the odds ratio of the combined end point (CEP) in patients with FH.

Indicator	Coefficient	Statistical Error	OR (Exp)	*p*
Deviation	−8.03	1.32	0.0003	<0.0001
Positive family history of CHD	2.16	0.48	8.67	<0.0001
Male gender	1.18	0.48	3.25	0.014
Age	0.08	0.02	1.08	<0.0001
Hypertension	1.07	0.46	2.92	0.021
Smoking	1.09	0.48	2.97	0.024

Note: It can be stated that increasing the age by 10 years leads to an increase in the OR (CEP) by 2.16 times. Formula for calculating OR and CEP probability: OR (CEP) = exp(−8.03 + 2.16 × Family history of CHD + 1.18 × male Sex + 0.08 × Age + 1.07 × Hypertension + 1.09 × Smoking. R (CEP) = 1/(1 + (1/OR)). The classification threshold = 0.45. If *p* (CEP) is <0.45, the CEP is not predicted. If *p* (CEP) is ≥0.45, the development of a CEP is predicted. The sensitivity is 67.6%; the specificity is 81.3%.

**Table 10:** Scale for assessing the risk of CEP developing in men with defined and probable familial hypercholesterolemia (FH).

Men (Risk CEP Minimum)						
Age < 30 years						
Positive family history	Yes	Yes	Yes	No	Yes	No
Hypertension	Yes	No	Yes	Yes	No	No
Smoking	Yes	Yes	No	Yes	No	No
Age 30–42 years						
Positive family history	Yes	Yes	Yes	No	Yes	No
Hypertension	Yes	No	Yes	Yes	No	No
Smoking	Yes	Yes	No	Yes	No	No
Age 43–56 years						
Positive family history	Yes	Yes	Yes	No	Yes	No
Hypertension	Yes	No	Yes	Yes	No	No
Smoking	Yes	Yes	No	Yes	No	No
Age 56–83 years						
Positive family history	Yes	Yes	Yes	No	Yes	No
Hypertension	Yes	No	Yes	Yes	No	No
Smoking	Yes	Yes	No	Yes	No	No
Age > 84 years						
Positive family history	Yes	Yes	Yes	No	Yes	No
Hypertension	Yes	No	Yes	Yes	No	No
Smoking	Yes	Yes	No	Yes	No	No

Note. Yes—the presence of a cardiovascular risk factors in the patient; no—the absence of a cardiovascular risk factors in the patient. Yellow background—high probability of CEP development (CEP development is predicted). Green background—low probability of CEP development (CEP development is not predicted).

**Table 11:** Scale for assessing the risk of combined end point developing in women with defined and probable FH.

Women (Risk CP Minimum)						
Age < 44 years						
Positive family history	Yes	Yes	No	Yes	No	No
Hypertension	Yes	No	Yes	No	No	No
Smoking	Yes	Yes	Yes	No	Yes	No
Age 44–57 years						
Positive family history	Yes	Yes	No	Yes	No	No
Hypertension	Yes	No	Yes	No	No	No
Smoking	Yes	Yes	Yes	No	Yes	No
Age 58–70 years						
Positive family history	Yes	Yes	No	Yes	No	No
Hypertension	Yes	No	Yes	No	No	No
Smoking	Yes	Yes	Yes	No	Yes	No
Age 71–84 years						
Positive family history	Yes	Yes	No	Yes	No	No
Hypertension	Yes	No	Yes	No	No	No
Smoking	Yes	Yes	Yes	No	Yes	No
Age ≥ 85 years						
Heredity	Yes	Yes	No	Yes	No	No
Hypertension	Yes	No	Yes	No	No	No
Smoking	Yes	Yes	Yes	No	Yes	No

Note. Yes—the presence of a cardiovascular risk factors in the patient; no—the absence of cardiovascular risk factors in the patient. Yellow background—high probability of CEP development (CEP development is predicted). Green background—low probability of CEP development (CEP development is not predicted).
